# Hsp90α promotes chemoresistance in pancreatic cancer by regulating Keap1-Nrf2 axis and inhibiting ferroptosis: Hsp90α promotes chemoresistance in PC

**DOI:** 10.3724/abbs.2024138

**Published:** 2024-08-22

**Authors:** Bin Liu, Zhiyuan Chen, Zhaoxing Li, Xinya Zhao, Weigang Zhang, Ao Zhang, Longxing Wen, Xiaoming Wang, Shuying Zhou, Daohai Qian

**Affiliations:** 1 Department of Hepatobiliary Surgery the First Affiliated Hospital of Wannan Medical College Wuhu 240001 China; 2 Pharmaceutical Research and Development Center School of Pharmacy Wannan Medical College Wuhu 240001 China; 3 School of Basic Medicine Wannan Medical College Wuhu 240001 China; 4 Department of Nursing Fudan University Shanghai Cancer Center; Department of Oncology Shanghai Medical College Fudan University Shanghai 200032 China

**Keywords:** heat shock protein 90α, Keap1-Nrf2 axis, ferroptosis, pancreatic cancer chemoresistance

## Abstract

Chemoresistance is the primary reason for poor prognosis in patients with pancreatic cancer (PC). Recent studies have indicated that ferroptosis may improve chemoresistance, but the underlying mechanisms remain unclear. In this study, significant upregulation of heat shock protein 90α (Hsp90α) expression is detected in the peripheral blood and tissue samples of patients with chemoresistant PC. Further studies reveal that Hsp90α promotes the proliferation, migration, and invasion of a chemoresistant pancreatic cell line (Panc-1-gem) by suppressing ferroptosis. Hsp90α competitively binds to Kelch-like ECH-associated protein 1 (Keap1), liberating nuclear factor erythroid 2-related factor 2 (Nrf2) from Keap1 sequestration. Nrf2 subsequently translocates into the nucleus and activates the glutathione peroxidase 4 (GPX4) pathway, thereby suppressing ferroptosis. This process further worsens the chemoresistance of PC cells. This study provides valuable insight into potential molecular targets to overcome chemoresistance in PC. It sheds light on the intricate mechanisms linking Hsp90α and ferroptosis to chemoresistance in PC and provides a theoretical foundation for the development of novel therapeutic strategies.

## Introduction

Pancreatic cancer (PC) is characterized by its highly malignant nature, rapid progression, and poor response to treatment, primarily due to chemotherapy resistance [1]. This resistance can arise from both intrinsic factors and acquired resistance mechanisms [2]. The precise mechanisms underlying gemcitabine-induced resistance in PC remain largely unknown, although previous studies have suggested that epithelial-mesenchymal transition, aberrant gene expression, gene mutation, dysregulation of critical signaling pathways, such as the NF-κB and Akt signaling pathways, apoptosis, and the presence of stromal cells, highly resistant cells, and cancer stem cells are involved in the chemoresistance of PC [ 3‒ 6].

Recently, heat shock protein 90α (Hsp90α) has emerged as a key player in the chemoresistance of PC [7]. Elevated level of Hsp90α has been shown to increase the anti-apoptotic capacity of tumor cells and contribute to chemoresistance [7]. We revealed how Hsp90α promotes the chemoresistance of PC by inhibiting ferroptosis, the exact details of which have not yet been elucidated.

Ferroptosis is a recently discovered non-apoptotic regulatory cell death mode characterized by lipid peroxidation due to excessive intracellular iron deposits and the accumulation of reactive oxygen species (ROS) [8]. It differs from apoptosis in terms of both morphology and mechanism. Apoptosis is a normal type of programmed cell death that can regulate tissue development and maintain homeostasis. The morphological features of apoptotic cells include reduced cell volume, damaged cell membranes, and chromatin condensation within the nucleus. In contrast, ferroptosis is characterized by mitochondrial atrophy, increased mitochondrial membrane density and thickness, decreased mitochondrial membrane cristae, and normal nuclear size with no chromatin concentration [9]. Glutathione peroxidase 4 (GPX4) plays a crucial role in eliminating detrimental lipid peroxides and preventing intracellular ferroptosis, thereby maintaining normal cellular function.

Although previous studies have linked ferroptosis to tumor resistance [ 9‒ 11], the specific mechanisms involved in the chemoresistance of PC remain poorly understood. In the present study, we revealed that Hsp90α competitively binds to Kelch-like ECH-associated protein 1 (Keap1), resulting in nuclear factor erythroid 2-related factor 2 (Nrf2) translocation into the nucleus, thereby regulating GPX4 expression [12]. Through this mechanism, Hsp90α inhibits ferroptosis and induces chemotherapy resistance in PC.

## Materials and Methods

### Study population and ethical statement

We retrospectively analyzed patients with PC who underwent chemotherapy at the Department of Hepatobiliary Surgery, Yijishan Hospital, Affiliated to Wannan Medical College, from June 2018 to November 2022. Patients were categorized into the sensitive group or the resistant group on the basis of common efficacy-related indicators such as complete response (CR), partial response (PR), progressive disease (PD), and stable disease (SD). Peripheral blood samples were collected to measure the levels of Hsp90α. For the resistant group, data were collected at the time of tumor enlargement or metastasis before a new intervention.

The inclusion criteria were as follows: (1) patients with a confirmed diagnosis of PC who received gemcitabine as a first-line chemotherapy drug after surgical resection or biopsy confirmation; (2) Tumor biomarker testing and imaging, including ultrasound, contrast-enhanced CT, or MRI, were conducted before each cycle of chemotherapy; and (3) Dynamic monitoring of relevant values in both sensitive and resistant states for the same individual.

The exclusion criteria were as follows: (1) patients who did not receive any treatment after the diagnosis of PC; and (2) Patients who did not complete full treatment cycles, making it impossible to evaluate treatment efficacy.

This study strictly adhered to the Helsinki principles and was approved by the Ethics Committee of Yijishan Hospital, Affiliated to Wannan Medical College. Written informed consents were obtained from all patients before their enrolment in the study.

### Immunohistochemistry (IHC)

The tissue samples were fixed in 4% paraformaldehyde for 3–4 h. Tissues with appropriate size were placed in embedding cassettes and sequentially processed through dehydration steps using different concentrations of alcohol and absolute ethanol. We subsequently immersed the dehydrated tissues and embedded cassettes in a tissue processor for paraffin infiltration. We pipetted liquid paraffin into the mold, retrieved the tissues from the embedding cassettes via forceps, and carefully placed them into the mold. The mold was covered, and a small amount of liquid paraffin was added and allowed to solidify on a cooling platform. Next, we used a microtome to section the cooled tissue samples and carefully transferred the sections to a water bath. The sections, together with the staining frame, were put into the repair solution for about 2 min after boiling and repairing, cool naturally and then put into an incubation box. Then 3% H _2_O _2_ was added dropwise and the sections were incubated at room temperature for 20 min. After the sections were rinsed with phosphate buffered saline (PBS, C10010500BT; Gibco, Carlsbad, USA), Hsp90α antibody (1:200, 60318-1-lg; Proteintech, Wuhan, China) was added and incubated at 37°C for 60 min. The sections were rinsed 3 times with PBS and incubated with the secondary antibody (1:200, PV-6000; Zsbio, Beijing, China) at 37°C for 20 min. The sections were rinsed again with PBS, and then DAB (ZLI-9018; Zsbio) chromogen was added and the color development was terminated after observing the effect, and subsequently hematoxylin staining and differentiation was performed. After staining, dehydration, clearing, and coverslipping were performed. Using a microscope (Nikon, Tokyo, Japan), we observed the locations of positive staining, indicated by brown color.

### Cell culture and drug-resistant cell line construction

PANC-1, Aspc-1, BxPc-3, and SW1990 cells obtained from the Cell Bank of the Chinese Academy of Sciences (Shanghai, China). PANC-1 was cultured in Dulbecco’s modified Eagle’s medium (DMEM; Gibco) supplemented with 1% antibiotics and 10% fetal bovine serum (FBS; Gibco). Aspc-1 and BxPc-3 were cultured in RPMI-1640 medium (Gibco). The cells were incubated at 37°C with 5% CO _2_ in a humidified incubator. SW-1990 was cultured in L-15 medium (Gibco) under a fully closed environment. A low-concentration gradient method was employed to construct a drug-resistant PC cell line. A mixture of 10 mL of DMEM and 400 μL gemcitabine (HY-17026; MedChemExpress, Monmouth Junction, USA) was prepared in a 15-mL centrifuge tube to reach a final gemcitabine concentration of 400 μM. Logarithmically growing PANC-1 cells were added to the suspension, and cell viability was monitored. The culture medium was discarded, and the cells were washed with PBS and replenished with fresh medium every week for 4 weeks. After stable growth and passage, the cells were cultured in DMEM supplemented with 450 μM gemcitabine. This process was repeated, and after 6 months, the gemcitabine-resistant cell line Panc-1-gem was obtained ( Supplementary Figure S1). The cell line was continuously passaged for subsequent experiments. When the cell density reached 70%–80%, logarithmically growing cells were harvested, subjected to trypsin digestion, and used for further experiments.

### Quantitative real-time PCR (qRT-PCR)

Logarithmically grown cells were suspended in 1 mL of Trizol reagent (TIANGEN, Beijing, China) to lyse the cells, followed by thorough mixing and a 5-min incubation at room temperature. Then, 200 μL of BCP reagent was added to the mixture, which was vigorously vortexed for 15 s and incubated at room temperature for 2–3 min. The mixture was centrifuged at 12,000 *g* and 4°C for 15 min, and the upper aqueous phase was carefully transferred to a new 1.5-mL EP tube. Then, 500 μL of isopropanol (KESHI, Chengdu, China) was added to the aqueous phase, mixed, and incubated at room temperature for 10 min. The mixture was then centrifuged again at 12,000 *g* and 4°C for 10 min, after which the supernatant was discarded. The RNA pellet was washed with 1 mL of 75% ethanol, followed by centrifugation at 7500 *g* and 4°C for 5 min. The ethanol in the supernatant was gently removed, and the RNA pellet was air-dried in an open EP tube until completely dry. The dried RNA was subsequently dissolved in 30 μL of DEPC-treated water, and the concentration was measured via a spectrophotometer. The primers for quantitative PCR were designed via Primer Premier 5.0 and Beacon Designer 7.8 software (primer sequences are provided in the Supplementary Table S1) and synthesized by Sangon Biotech (Shanghai, China). Reverse transcription was performed via the appropriate reaction system. The relative expression level of mRNA was calculated via the 2 ^–ΔΔCt^ method on the basis of the Ct values.

### Western blot analysis

Cells in the logarithmic growth phase were seeded at a density of 1 × 10 ^5^ cells/mL in a 6-well plate, with 2 mL of medium per well. After grouping and treatment, the cells were harvested. Total protein extraction was performed by adding protein lysis buffer containing 1% PMSF to each group. The cells were then lysed on ice for 30 min, followed by centrifugation at 13,400 *g* for 15 min. The supernatant was collected for protein quantification, and 5 ×  sample buffer (1/4 of the supernatant volume) was added. The mixture was boiled at 100°C for denaturation. A 10% separating gel and a 6% stacking gel were prepared. Then, 30 μg of total protein was loaded into each well, and electrophoresis was performed at 70 V for 30 min, followed by 110 V for 90 min. The proteins were subsequently transferred onto a PVDF membrane (Millipore, Billerica, USA) at a constant current of 200 mA for 120 min. After being blocked with 5% skim milk powder at room temperature for 2 h, the membrane was incubated with primary antibodies at 4°C overnight and then with corresponding second antibody. The following day, the membrane was washed with TBST 3 times (10 min each). ECL detection reagent was added, and the protein bands were visualized via an imaging system. The grayscale values were analyzed via ImageJ 1.43 software. The primary antibodies against the following proteins were used: Hsp90α (1:2000; Proteintech), GPX4 (1:2000; Abcam, Cambridge, UK), Nrf2 (1:2000; Proteintech), Keap1 (1:2000; Proteintech) and GAPDH (1:1000; CST, Danvers, USA). The secondary antibody was as follows: AffiniPure Goat Anti-Rabbit IgG (H  +  L) (1:2000; Boster, Wuhan, China).

### ELISA

The ELISA kit (PROTGEN, Yantai, China) was equilibrated at 37°C for 30 min. The concentrated washing solution was mixed with 475 mL of deionized water, and the mixture was set aside. For the calibration standard, 0.4 mL of the analyte dilution buffer was added. The cell culture supernatant was diluted 20 times. The required number of strips was placed on the plate holder, and a calibration standard and sample wells were set up. Then, 50 μL of the calibration standard and diluted samples were added to each well. Thereafter, 50 μL of Hsp90α detection reagent was added to each well and gently mixed. The plate was covered with a sealing film and incubated at 37°C for 60 min. After the reaction mixture was discarded, the plate was washed with 300 μL of washing solution 6 times. Finally, the plate was inverted and dried on absorbent paper. Then, 50 μL of chromogenic reagents A and B were added to each well, gently mixed, and incubated at 37°C for 20 min. Subsequently, 50 μL of stop solution was added to each well to terminate color development. The OD value was read at 450 nm within 10 min. The standard curve was plotted via the software provided with the instrument, and the OD value of the test sample was substituted into the regression equation to calculate the amount of Hsp90α.

### Drug concentration screening

Nrf2 inhibitor (ML385; MedChemExpress), the ferroptosis inhibitor (Ferrostatin-1, Fer-1; MedChemExpress), Hsp90α inhibitor (17-DMAG; MedChemExpress) were used in this experiment. DMSO was used to dissolve the exogenous drugs into a high-concentration stock solution and set up a gradient of concentration based on this, and at the same time, we used cytotoxicity assay to detect the viability of the cells at different concentrations and different time points, in order to select the most suitable drug for use at different time. We also used cytotoxicity assay to test the cell viability at different concentrations and time, in order to screen out the optimal time and concentration of the drug.

### Cell proliferation assay (CCK8)

Under sterile conditions, a single-cell suspension was prepared by digesting the cells with trypsin and centrifuging them at 300 *g* for 3 min at room temperature. The cells were then counted, and the density was adjusted to 1 × 10 ^4^ cells/mL. Next, 100 μL of the cell suspension was added to each well of a 96-well plate. The plate was incubated in a CO _2_ incubator at 37°C for 24 h. After incubation, 10 μL of different concentrations of the test substance was added to each well. The plate was further incubated for 24 h. Next, 10 μL of CCK8 solution (Bestbio, Shanghai, China) was added to each well, and the plate was incubated for 1–4 h at 37°C. The absorbance was then measured at 450 nm via an ELISA reader (BioTek, Vermont, USA).

### Flow cytometry

When the cell confluence of each experimental group reached approximately 70%, apoptosis was induced by the drug. Both adherent cells and cells from the supernatant were collected. The cells were detached with trypsin, and a single-cell suspension was prepared. The cells from the supernatant were collected in the same 5 mL centrifuge tube. Each group had three replicate wells, and the cells were centrifuged at 400 *g* for 5 min. The supernatant was discarded, and the cell pellet was washed with prechilled D-Hanks buffer (pH = 7.2‒7.4) at 4°C. The cell pellet was then washed once with 1 ×  binding buffer and centrifuged at 400 *g* for 3 min, after which the cells were collected. The cell pellet was resuspended in 200 μL of 1 ×  binding buffer. Subsequently, 2 μL of Annexin V-APC and PI staining solution was added, and the mixture was incubated in the dark at room temperature for 20–60 min. Depending on the cell quantity, 200–300 μL of 1 ×  binding buffer was added, and the samples were analyzed using a flow cytometer.

### Wound healing assay

The cell line of interest was cultured until it reached the logarithmic growth phase and then seeded at an appropriate density in a culture dish or multi-well plate. A grid-like scratch was created on the surface of the culture dish using a tool. A 200-μL pipette tip was then gently moved across the wound area. The scratched cell layer was washed gently with serum-free culture medium or PBS to remove debris and detached cells. The culture dish was replenished with an appropriate culture medium, allowing the cells to continue growing. The behavior of the cells at the scratch site was observed under a microscope, and the migration process was recorded at 0 h and 24 h. Finally, the degree of scratch area reduction was measured and compared using ImageJ software to evaluate the extent of cell migration.

### Transwell experiment

The reagent kit was taken from a –20°C refrigerator, and the required inserts were placed into a new 24-well plate. The plate was placed on a sterile workbench, and the inserts were allowed to equilibrate to room temperature. Then, 500 μL of serum-free culture medium was added to the upper and lower chambers of each insert and incubated in a 37°C cell culture incubator for 2 h to rehydrate the Matrigel matrix layer. A serum-free cell suspension was prepared, and the number of cells was counted. Typically, 10 ^5^ cells per well (in a 24-well plate) were used. Once the Matrigel matrix layer was rehydrated, we transferred the inserts to a new plate, carefully removed the culture medium from the upper chamber, and added 100 μL of cell suspension. In the lower chamber, 600 μL of 10% FBS culture medium was added, and the mixture was incubated for a specific period of time at 37°C. The inserts were inverted onto absorbent paper to remove the culture medium. Non-transferred cells were gently removed from the upper chamber using a cotton swab, and the inserts were washed three times with PBS. Each insert was fixed with 4% paraformaldehyde for 30 min. The inserts were then inverted onto the lid of a 24-well plate, and the bottom surface was air-dried in a fume hood for approximately 30 min. Another 24-well plate (without inserts) was used, 500 μL of 0.1% crystal violet was added, and the inserts were placed in the plate. The membrane was submerged in the solution and then incubated in a cell culture incubator for 30 min. Afterward, the inserts were removed and washed with PBS three times, and images were captured. Images were taken by randomly selecting the fields of view for each transwell insert. Three photographs per well were taken at 100 ×  magnification, and the number of cells was counted.

### Transfection and lentiviral knockdown

siRNA sequences were designed and synthesized by Sangon Biotech for Hsp90α (si-Hsp90α) and Nrf2 (si-Nrf2) ( Supplementary Table S2). The overexpression plasmids and knockdown lentiviral vectors were constructed by Bioultra Ltd. (Heifei, China). For transfection, logarithmically growing cells were seeded at a density of 1 × 10 ^5^ cells/mL in 6-well plates. When the cell density reached approximately 50%, we performed cell transfection. Then, 2 μL of Lipo 3000 transfection reagent was mixed with 125 μL of reduced serum culture medium, and 2.5 μL of Lipo 3000 transfection reagent was mixed with 2.5 μL of plasmid. Then, the prepared transfection reagent was mixed with the corresponding overexpression plasmid for 15 min before this mixture was added to 6-well plates. The mixture was incubated in a cell culture incubator for 6 h, after which the medium was replaced. After 24 h, the experimental treatment was performed, and relevant assays were performed after 24 h of treatment. For lentiviral knockdown, 1 × 10 ^5^ cells/well were seeded in 6-well plates and incubated overnight. The cells were transduced with 6 × 10 ^6^ lentiviral particles/well. We selected stable clones via puromycin for 2 weeks. To determine the transfection efficiency, qRT-PCR and western blot analysis were employed.

### Tumorigenicity in nude mice

All animal experiments were conducted following the protocol approved by the Laboratory Animal Welfare and Ethics Committee of Wannan Medical College. The experiments complied with the ARRIVE guidelines and the National Research Council’s “Guide for the Care and Use of Laboratory Animals”. Nude mice were randomly assigned to the control group, shNC group, or shHsp90α group, with 6 mice in each group. Sufficient cells were prepared. Tumor cells in the logarithmic growth phase were digested with trypsin and resuspended. The cells were counted and resuspended in a specific volume of PBS to achieve a cell concentration of 5 × 10 ^7^ cells/mL. Following cell preparation, a disposable sterile syringe was used to collect the cells, which were then subcutaneously injected into the animals at 200 μL per mouse. After 24 days, tumor formation was observed, and tumors larger than 5 mm in diameter were selected. The mice received intraperitoneal administration of gemcitabine twice a week, and the tumor size and animal body weight were measured. Data were collected for 8 measurements. On day 49 after subcutaneous injection, the animals were euthanized via an overdose of 10% chloral hydrate, followed by cervical dislocation to confirm death. The animals were then arranged on a scaled whiteboard. Specific measurements were observed and recorded via a digital camera (Nikon). Tumors were excised via surgical scissors and forceps, arranged on a scaled whiteboard, and photographed for documentation.

### Sequencing analysis

The experimental subjects were divided into two groups: the Panc-1 group and the Panc-1-gem group. Cells in the logarithmic growth phase from both groups were collected. Total RNA was extracted from the cell suspension using 1 mL of Trizol. The extracted samples were labelled according to the protocol and sent to Novo Company (Beijing, China) for sequencing analysis. GSEA and KEGG analysis techniques were used to analyze differential gene and metabolic pathway enrichment. Data analysis was performed on the Novo Cloud Platform.

### Transmission electron microscopy

Cells growing on the adherent culture plate were gently taped into a 6-well culture plate. Then, the cells were transferred from the culture plate to a centrifuge tube and centrifuged at 250 *g* for 8–10 min. The supernatant was carefully aspirated. The cell pellets were transferred into small vials and rinsed with PBS 2 times (15 min each). Then, the cells were fixed with 3% glutaraldehyde solution at 4°C overnight. The fixative was removed, and the cells were rinsed with PBS 3 times (10 min each). The cells were fixed with 1% osmium tetroxide at 4°C for 30 min, followed by rinsing with PBS 3 times. Dehydration was performed via the addition of a series of increasing concentrations of acetone, including 50%, 70%, 90%, and 100% (10 min each). The dehydration reagent was removed, and a diluted embedding agent was added and incubated for 30 min. The diluted embedding agent was discarded, and the pure embedding agent was added and left at room temperature for 2 h. Then, the cell pellet was transferred to the capsule module filled with mixed embedding agent, filled with the embedding agent, and placed in a 60°C oven for 2 h. We used an ultramicrotome to cut 70-nm-thick ultrathin sections and placed them on dental wax slices in a clean culture dish. The sections were then soaked with lead citrate staining solution, and the culture dish was covered. After 5–30 min of staining, the sections were immediately washed with double distilled water 3 times. Excess moisture was removed, and the sections were naturally dried. The aforementioned steps were repeated, and the grid was placed in another culture dish with wax slices for lead citrate staining and washing. After air drying, we took photographs via transmission electron microscopy.

### Lipid ROS detection

A storage solution of 10 mM BODIPY 581/591 C11 (Cat#D3861; Thermo Fisher Scientific, Waltham, USA) was prepared before the experiment. Target cells of suitable density were cultured and treated appropriately to establish relevant cell models as needed for the experiment. The cells were incubated for 1 h after the appropriate concentration of BODIPY 581/591 C11 was added to reach a final concentration of 5 μM. After incubation for flow cytometry, the cells were washed three times with PBS to remove excess dye. Then, trypsin was added to digest the experimental target cells, after which they were resuspended in PBS containing 5% FBS, followed by flow cytometry with excitation at 488–565 nm. The corresponding signals were measured at 505–550 nm in the FL1 channel and at more than 580 nm in the FL2 channel.

### Iron concentration detection

Logarithmic phase shCtrl and shHsp90α cells were inoculated into a 96-well plate at a density of 5 × 10 ^7^ cells/L. The cell supernatant was collected and centrifuged at 1000 *g* for 10 min to remove particles and aggregates. After 48 h of incubation, we discarded the old culture medium and washed the cells with serum-free medium three times. We prepared a working solution of 1 μM by diluting a 1 M FerroOrange solution (Abcam, Cambridge, UK) at a ratio of 1:1000. Then, 200 μL of FerroOrange working solution was added to the cells, which were subsequently incubated in a 5% CO _2_ incubator at 37°C for 30 min. The absorbance was read at a wavelength of 546 nm for colorimetric measurement. We calculated the iron concentration (μM) using the following formula: iron concentration=[(OD _total iron_‒OD _blank_)/(OD _calibrator_‒OD _blank_)] × 71.6.

### Immunofluorescence staining

The logarithmic phase cells were collected and washed twice with PBS by centrifugation (300 *g*, 5 min). The cell smears were directly prepared. The cells were fixed with an appropriate fixative according to the specific requirements. The cells were washed with PBS three times (5 min each). The cells were fixed with 4% paraformaldehyde for 30 min and then washed with PBS three times. The cells were permeabilized with 0.1% Triton X-100 at 25°C and washed with PBS three times. The cells were blocked for 30 min using blocking solution, subsequently the sections were subjected to incubation with a primary antibody against Hsp90α (1:1000; Affinity, New York, USA), Nrf2 (1:1000; Affinity), Keap1(1:1000; Affinity). We then used a secondary antibody conjugated with green (1:1000, BA1127; BOSTER, Wuhan, China) or red (1:1000, BA1133; BOSTER) fluorescent dye. After the addition of an anti-fluorescence quencher and DAPI staining, the cells were observed with a fluorescence microscope.

### Protein nucleocytoplasmic separation

We mixed the three reagents at room temperature to ensure their even distribution and then set them aside on ice. We took an appropriate amount of cell lysate and protein extraction reagent A and added 1 mM PMSF to the mixture a few minutes before use. The adherent cells were washed once with PBS and then gently agitated with a cell scraper. The cell suspension was centrifuged, and the supernatant was carefully removed. Then, 20 μL of the cell pellet was combined with 200 μL of cell lysate protein extraction reagent A containing PMSF. To fully suspend and disperse the sample, the mixture was vigorously vortexed at the highest speed for 5 s. The samples were placed on ice for 10–15 min. After that, 10 μL of cell lysate protein extraction reagent B was added. The mixture was vortexed again at the highest speed for 5 s, after which the samples were kept in an ice bath for 1 min. The vortexing step was repeated at the highest speed for 5 s. The samples were subsequently centrifuged at 12,000–16,000 *g* and 4°C for 5 min. The supernatant was carefully transferred to pre-cooled plastic tubes to obtain the extracted cell lysate protein. The same procedure was used to isolate the cell nuclear proteins. For the cell pellet, we completely removed any residual supernatant before adding 50 μL of cell nuclear protein extraction reagent containing PMSF. The mixture was vigorously vortexed for 15–30 s. The samples were returned to the ice bath and vortexed every 1–2 min for 30 min. The samples were subsequently centrifuged at 12,000–16,000 *g* and 4°C for 10 min. The supernatant was immediately transferred to pre-cooled plastic tubes to obtain the extracted nuclear proteins.

### Co-immunoprecipitation (Co-IP) assay

Panc-1-gem cells were transfected with the Flag-Hsp90α plasmid, and the cells were harvested 48 h after transfection to extract total protein. The cells were divided into input, Flag (experimental group) and IgG (negative control) groups, the experiments were conducted following the instructions of the Co-IP kit (Thermo Fisher Scientific), and the obtained protein precipitates were used for the subsequent experiments.

### Statistical analysis

Statistical analysis of intergroup differences was performed via Student’s *t* tests. Data are expressed as the mean ± SD. If the variances were homogeneous, analysis of variance (ANOVA) was applied. For data with non-homogeneous variances, a rank transformation was performed to achieve homogeneity of variances, and subsequently, ANOVA was used for analysis. Data analysis was conducted using SPSS 22.0 software, with the significance threshold set at *P*  < 0.05.

## Results

### Hsp90α was upregulated in the peripheral blood and tissue samples of patients with chemotherapy-resistant PC and Panc-1-gem cells

**Figure 1:**
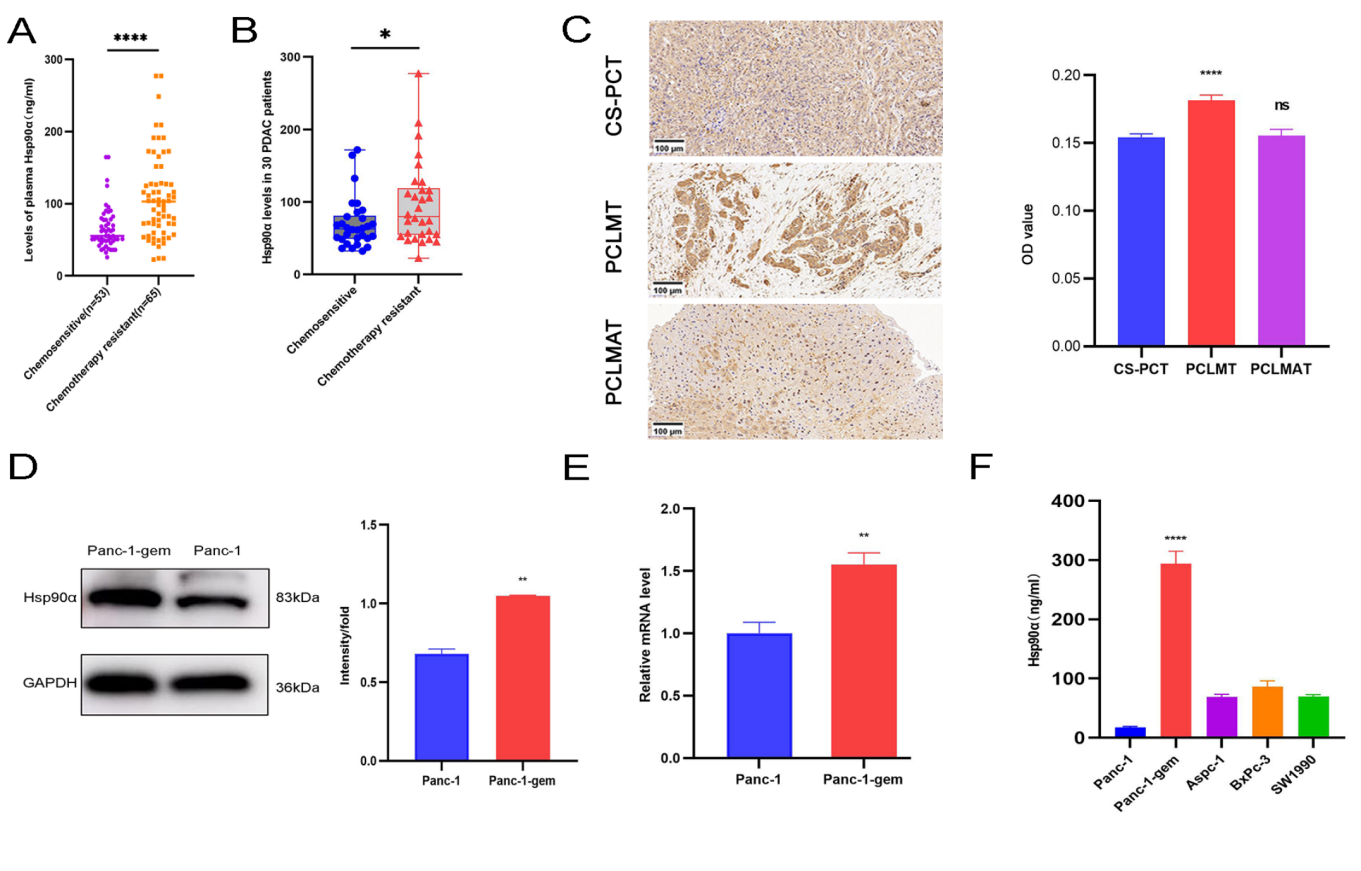
Figure 1 Hsp90α was upregulated in peripheral blood and tissue samples of patients with chemotherapy-resistant PC and Panc-1-gem cells (A) The levels of Hsp90α in peripheral blood of PC patients with chemosensitive (n = 53) and chemoresistant (n = 65). (B) The levels of Hsp90α in peripheral blood of the same PC patients who were initially chemosensitive and then gradually developed to chemoresistance (n = 30). (C) IHC staining of PCT in chemosensitive PC and PCLMT/PCLMAT in chemoresistant PC. Scale bar: 100 μm. (D) Western blot analysis of Hsp90α protein in Panc-1-gem and Panc-1 groups. (E) Relative mRNA level of Hsp90α in Panc-1-gem and Panc-1 groups. (F) ELISA was used to detect Hsp90α in the supernatant of Panc-1-gem, Panc-1, Aspc-1, BxPc-3, and SW1990 groups. Data are presented as the mean ± SD. **P < 0.01, ****P < 0.0001.

We observed that peripheral blood Hsp90α level was significantly higher in the chemoresistant group ( *n*  = 65) than in the chemosensitive group ( *n*  = 53) ( Figure 1A). Furthermore, we dynamically followed patients to monitor the peripheral blood level of Hsp90α during the transition from the chemosensitive stage to the chemoresistant stage ( *n*  = 30). Hsp90α level was significantly elevated in the chemoresistant stage compared with that in the sensitive stage ( Figure 1B).

Immunohistochemical analysis revealed that the expression of Hsp90α in chemosensitive PC tissues (CS-PCT) was not significantly different from that in surrounding tissues or PC liver metastasis adjacent tissues (PCLMAT). The presence of liver metastases from PC indicates the development of chemoresistance. In the chemoresistance stage, the expression of Hsp90α was significantly higher in the tissue samples of patients with PC liver metastasis tissue (PCLMT) than in those of patients with CS-PCT and PCLMAT ( Figure 1C).

On the basis of these findings, we successfully established a chemoresistant strain of PC (Panc-1-gem) ( Supplementary Figure S1). Further studies revealed that both the protein ( Figure 1D) and mRNA ( Figure 1E) levels of Hsp90α were higher in the Panc-1-gem cell line than in the Panc-1 cell line. Additionally, ELISA-based quantitative analysis of cell culture supernatants revealed significantly higher levels of Hsp90α in Panc-1-gem cells than in Panc-1, Aspc-1, BxPc-3, and SW1990 cells ( Figure 1F).

### Hsp90α facilitated proliferation and invasion while inhibiting the apoptosis of Panc-1-gem cells

**Figure 2:**
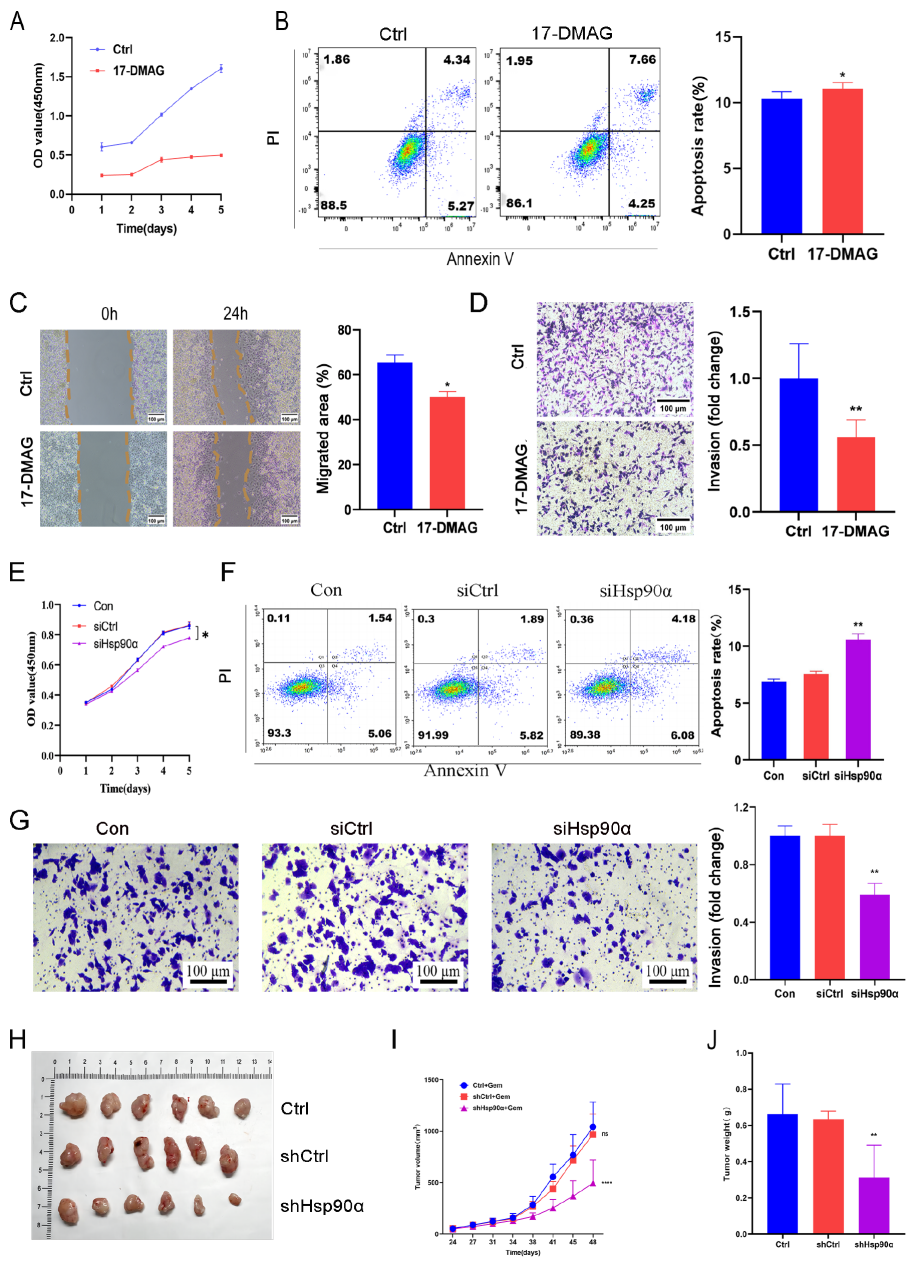
Figure 2 Hsp90α facilitated proliferation and invasion while inhibiting apoptosis of Panc-1-gem cells (A) Cell Counting Kit-8 (CCK8) of Ctrl and 17-DMAG groups. (B) Flow cytometry of Ctrl and 17-DMAG groups. (C) Wound healing assay of Ctrl and 17-DMAG groups, scale bar: 100 μm. (D) Transwell assay of Ctrl and 17-DMAG groups. Scale bar: 100 μm. (E) Cell Counting Kit-8 (CCK8) of Ctrl, siCtrl and siHsp90α groups. (F) Flow cytometry of Ctrl, siCtrl and siHsp90α groups. (G) Transwell assay of Ctrl, siCtrl and siHsp90α groups. Scale bar: 100 μm. (H) The tumor images, volume quantification and weight measurement in the Ctrl + gem, shCtrl + gem, and shHsp90α + gem groups. Data are presented as the mean ± SD. *P<0.05, **P<0.01, ****P<0.0001.

Panc-1-gem cells treated with 50 nM Hsp90α inhibitor (17-DMAG) ( Supplementary Figure S2A,B) were added as an experimental group, while a blank control group was set up. The cell proliferation of the 17-DMAG and Ctrl groups was observed continuously for 5 days, and the results revealed that 17-DMAG decelerated the proliferation of Panc-1-gem cells ( Figure 2A) and increased the Panc-1-gem cell apoptosis rate ( Figure 2B). Wound healing and Transwell assays indicated that 17-DMAG reduced Panc-1-gem cell migration ( Figure 2C) and invasion ( Figure 2D). Moreover, we targeted Hsp90α via siRNA interference ( Supplementary Figure S2C,D). Compared with the Ctrl and siCtrl groups, the siHsp90α group exhibited significantly diminished proliferation ( Figure 2E), increased apoptosis ( Figure 2F), and a reduced invasion rate ( Figure 2G).

Additionally, murine xenograft experiments revealed that after 48 days of tumor growth, the shHsp90α group presented significantly lower tumor volume and weight than the Ctrl and shCtrl groups ( Figure 2H).

### Ferroptosis was inhibited in Panc-1-gem cells

**Figure 3:**
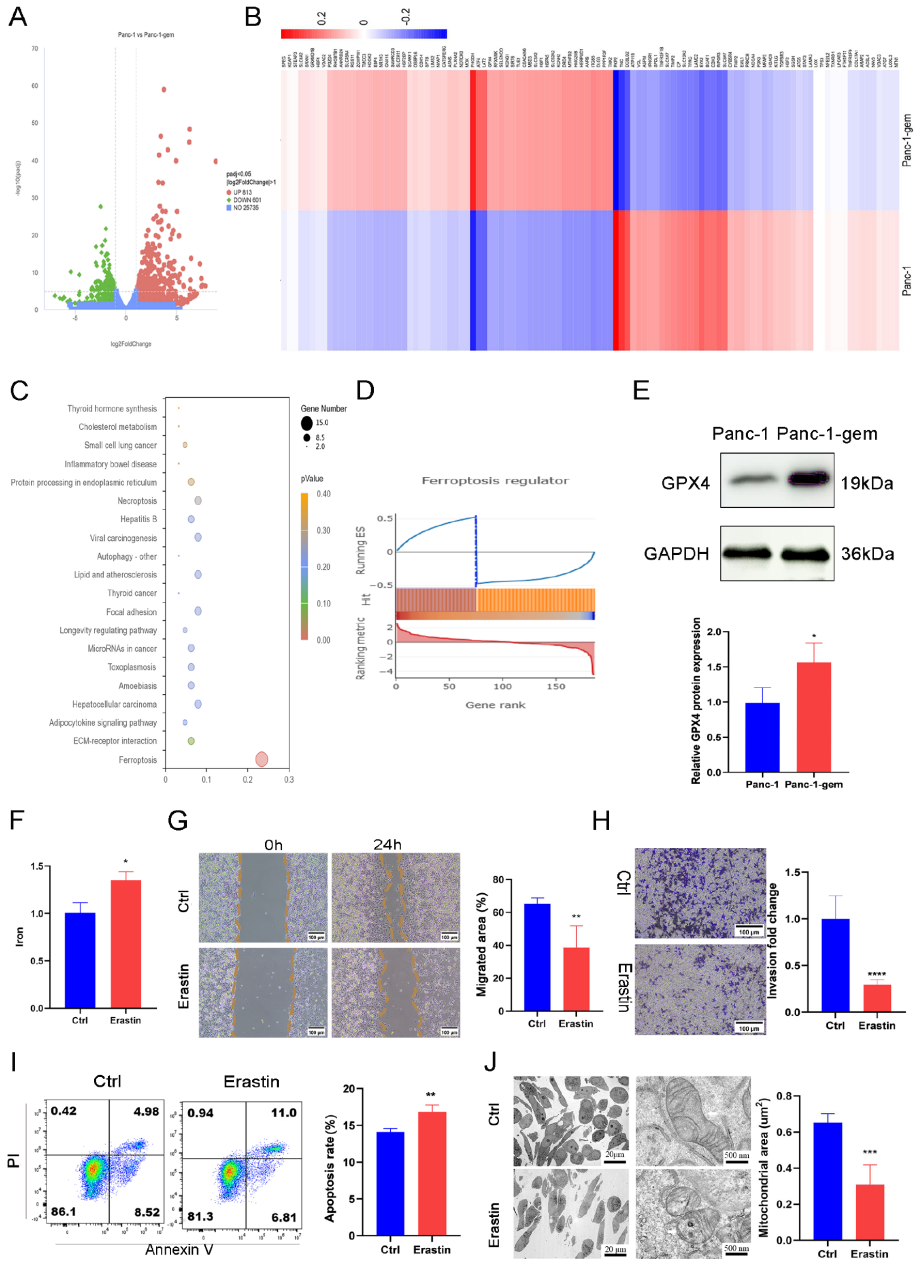
Figure 3 Ferroptosis was inhibited in Panc-1-gem cells (A) The volcano plot showingthe differential gene expression between Panc-1 and Panc-1-gem groups. (B) The heat map illustrating differential gene expression between Panc-1 and Panc-1-gem groups. (C) The pathway Enrichment Map (KEGG) of Panc-1 and Panc-1-gem groups. (D) GSEA enrichment analysis of the ferroptosis pathway. (E) Relative expression and quantification of GPX4 protein of Panc-1 and Panc-1-gem groups. (F) The results of iron concentration measurement of the Panc-1-gem group after adding erastin. (G) Wound healing assay of Panc-1-gem cells after adding erastin. Scale bar: 100 μm. (H) The transwell assay results of cell invasion quantification in the Panc-1-gem group after adding erastin. Scale bar: 100 μm. (I) Flow cytometry of the Ctrl and erastin groups. (J) Transmission electron microscopy was used to observe cell morphology (scale bar: 20 μm); and mitochondrial morphology (scale bar: 500 nm of the Ctrl and erastin groups). Data are presented as the mean ± SD. *P < 0.05, **P < 0.01, ***P < 0.001, ****P < 0.0001.

Sequencing analysis revealed substantial differential regulation of genes associated with ferroptosis ( Figure 3A,B). Furthermore, the results of the KEGG enrichment analysis revealed significant differences in the ferroptosis pathway ( Figure 3C). GSEA enrichment analysis was subsequently performed to explore the correlation of ferroptosis with gene expression levels ( Figure 3D). Western blot analysis revealed increased expression of GPX4 in Panc-1-gem cells compared with that in Panc-1 cells ( Figure 3E). Subsequently, 5 μM erastin (a ferroptosis activator) (Supplementary Figure S3A, B) was added to Panc-1-gem cells. ELISA results revealed a notable increase in the intracellular iron concentration in the erastin group compared with the Ctrl group ( Figure 3F). Wound healing and Transwell assays revealed decreased cell migration ( Figure 3G) and invasion ( Figure 3H) in the erastin group. Additionally, the number of apoptotic cells was increased in the erastin group ( Figure 3I). Morphological changes, such as mitochondrial shrinkage and increased mitochondrial membrane density, were observed by transmission electron microscopy ( Figure 3J).

### Hsp90α inhibition induced ferroptosis in Panc-1-gem cells

**Figure 4:**
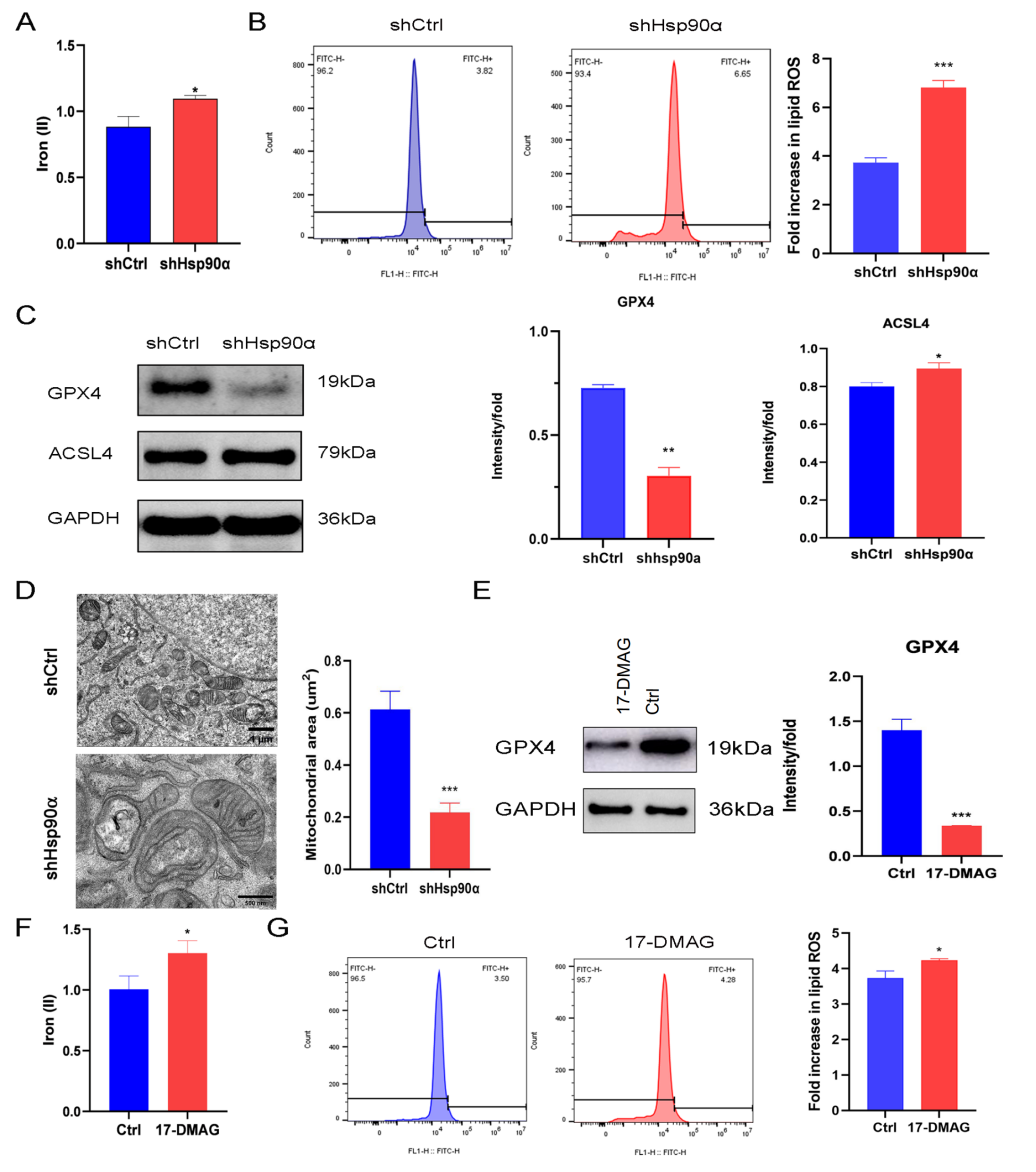
Figure 4 Hsp90α inhibition induced ferroptosis in Panc-1-gem cells (A) Quantitative detection of iron content in shCtrl and shHsp90α groups using iron reagent kit. (B) Measurement and quantitative analysis of lipid ROS content in shCtrl and shHsp90α groups. (C) Western blot analysis of GPX4 and ACSL4 protein expressions in shCtrl and shHsp90α groups. (D) Morphological changes of mitochondria in shCtrl (scale bar: 1 μm) and shHsp90α groups (scale bar: 500 nm) were observed by transmission electron microscope. (E) Western blot analysis of GPX4 protein expression in Ctrl and 17-DMAG groups. (F) Iron assay kit was used to detect iron contents in Ctrl and 17-DMAG groups. (G) Measurement and quantitative analysis of lipid ROS content in Ctrl and 17-DMAG groups. Data are presented as the mean ± SD. *P < 0.05, **P < 0.01, ***P < 0.001.

Compared with the control group, the shHsp90α group presented increased intracellular iron ( Figure 4A) and lipid ROS concentrations ( Figure 4B). Western blot analysis revealed a marked reduction in the expression level of GPX4 in the shHsp90α group compared with that in the shCtrl group. In contrast, ACSL4 expression was significantly increased in the shHsp90α group ( Figure 4C). Transmission electron microscopy revealed mitochondrial shrinkage and increased mitochondrial membrane density in Panc-1-gem cells treated with shHsp90α ( Figure 4D). Furthermore, compared with the control, 17-DMAG significantly reduced GPX4 expression ( Figure 4E), increased iron concentrations ( Figure 4F), and elevated lipid ROS levels ( Figure 4G) in Panc-1-gem cells.

### Nrf2 inhibition reduced the proliferation, invasion, and migration of Panc-1-germ cells and hindered ferroptosis

**Figure 5:**
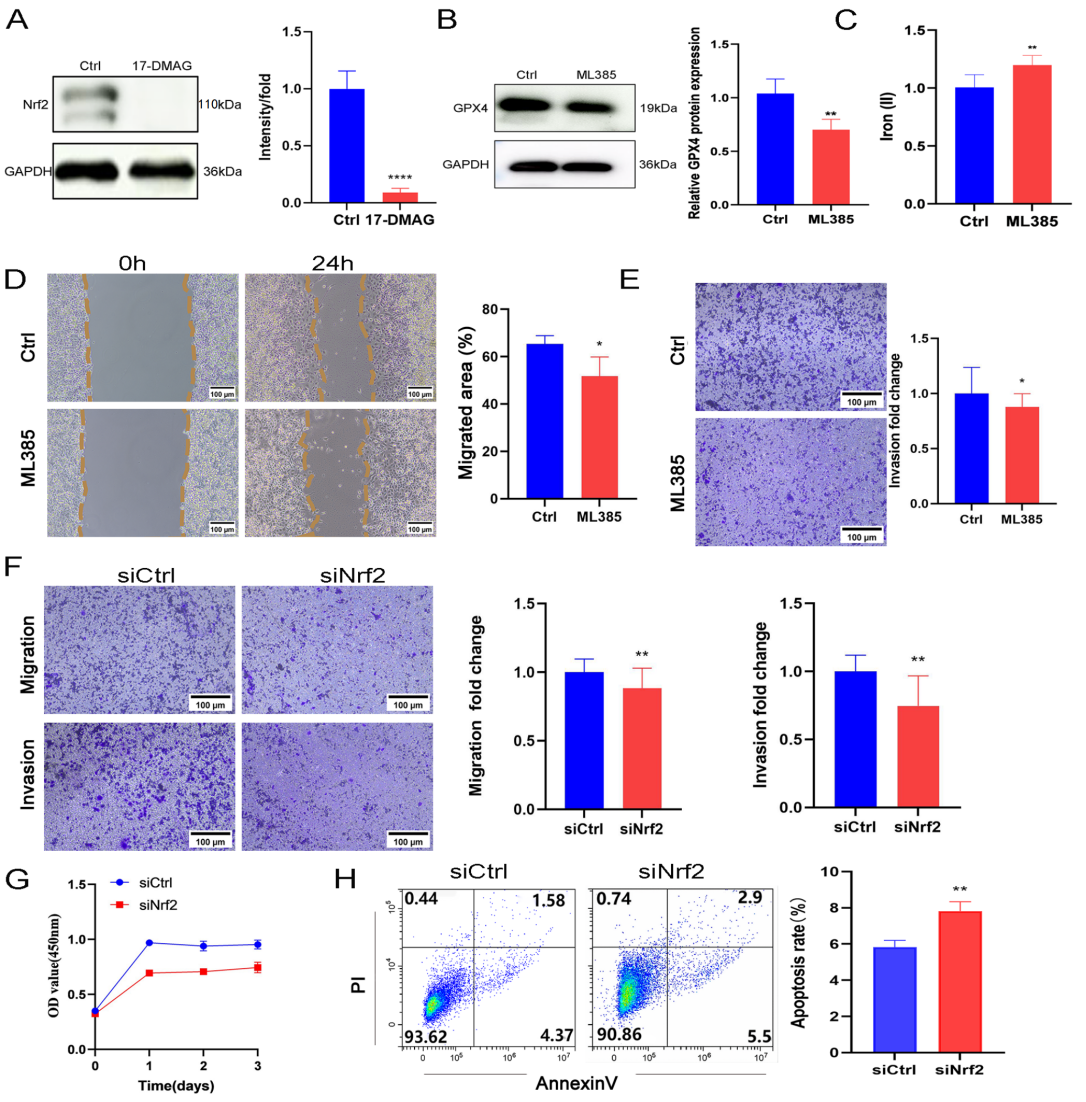
Figure 5 Nrf2 inhibition reduced the proliferation, invasion, and migration of Panc-1-germ cells, and hindered ferroptosis (A) Western blot analysis of the expression of Nrf2 in Ctrl and 17-DMAG groups. (B) Western blot analysis of GPX4 expression in Ctrl and ML385 groups. (C) Iron reagent kit was used for quantitative measurement of iron concentration in Ctrl and ML385 groups. (D) Wound healing assay of Ctrl and ML385 groups. Scale bar: 100 μm. (E) Transwell assay of Ctrl and ML385 groups. Scale bar: 100 μm. (F) Transwell assay of siCtrl and siNrf2 groups. Scale bar: 100 μm. (G) CCK8 assay of siCtrl and siNrf2 groups. (H) Flow cytometry analysis of apoptosis rate in Ctrl and siNrf2 groups. Data are presented as the mean ± SD. *P < 0.05, **P < 0.01, ****P < 0.0001.

After treating Panc-1-gem cells with 50 nM 17-DMAG, the results of western blot analysis revealed that the protein expression level of Nrf2 was significantly lower in the 17-DMAG group than in the control group ( Figure 5A). We subsequently added 0.4 μM of the Nrf2 inhibitor (ML385) ( Supplementary Figure S3C) to Panc-1-gem cells. Notably, the ML385 group presented reduced expression of GPX4 ( Figure 5B), increased intracellular iron concentrations ( Figure 5C), and weakened invasion and migration abilities ( Figure 5D,E). Furthermore, we employed the siRNA interference technique to validate our findings, and the results demonstrated that the siNrf2 group had diminished proliferation, migration, and invasion ( Figure 5F,G) and increased apoptosis ( Figure 5H).

### Competitive binding of Hsp90α to Keap1 facilitated the nuclear translocation of Nrf2, inducing GPX4 expression and inhibiting ferroptosis

**Figure 6:**
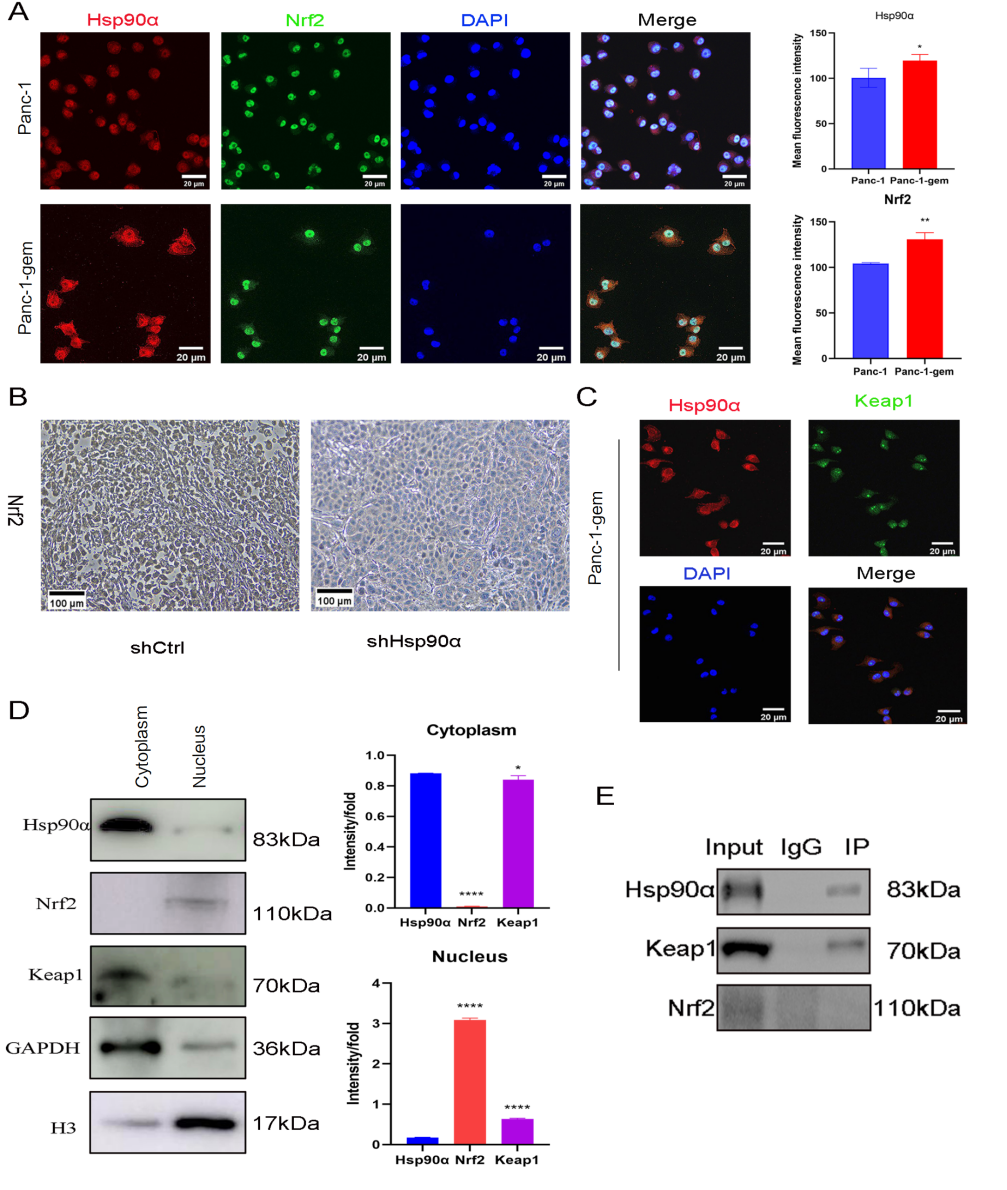
Figure 6 Competitive binding of Hsp90α to Keap1 facilitated nuclear translocation of Nrf2, inducing GPX4 expression and inhibiting ferroptosis (A) Immunofluorescence staining of Panc-1 and Panc-1-gem groups. Scale bar: 20 μm. (B) Immunohistochemical staining of Hsp90α and Nrf2 in shCtrl and shHsp90α groups. Scale bar: 100 μm. (C) Immunofluorescence staining of Hsp90α and Keap1 in the Panc-1-gem group. Scale bar: 20 μm. (D) Western blot analysis of Hsp90α, Nrf2, and Keap1 proteins in the cytoplasm and nucleus of Panc-1-gem cells. (E) Co-IP validation of Hsp90α, Keap1 and Nrf2 proteins. Data are presented as the mean ± SD. *P < 0.05, **P < 0.01, ****P < 0.0001.

Immunofluorescence staining revealed that the fluorescence intensity of Nrf2 in Panc-1-gem cells was greater in the nucleus, whereas the fluorescence intensity of Nrf2 in Panc-1 cells was greater in the cytoplasm ( Figure 6A). Immunohistochemical staining also revealed that Nrf2 was predominantly distributed in the cytoplasm of shHsp90α-treated tumor tissues, whereas in the shCtrl group, Nrf2 was predominantly localized in the nucleus ( Figure 6B). In addition, Keap1 was found to have the strongest fluorescence signal intensity in the cytoplasm of Panc-1-gem cells ( Figure 6C). Subsequent nuclear and cytoplasmic fractionation followed by western blot analysis revealed increased expressions of Hsp90α and Keap1 in the cytoplasm, whereas increased nuclear level of Nrf2 was detected in Panc-1-gem cells ( Figure 6D). Co-IP revealed the protein expressions of Hsp90α and Keap1 in both the Input and IP groups, but Nrf2 expression was not detected in the IP group ( Figure 6E).

### Synergistic effects of 17-DMAG, ML385, and Fer-1 in rescuing cell viability and proliferation

**Figure 7:**
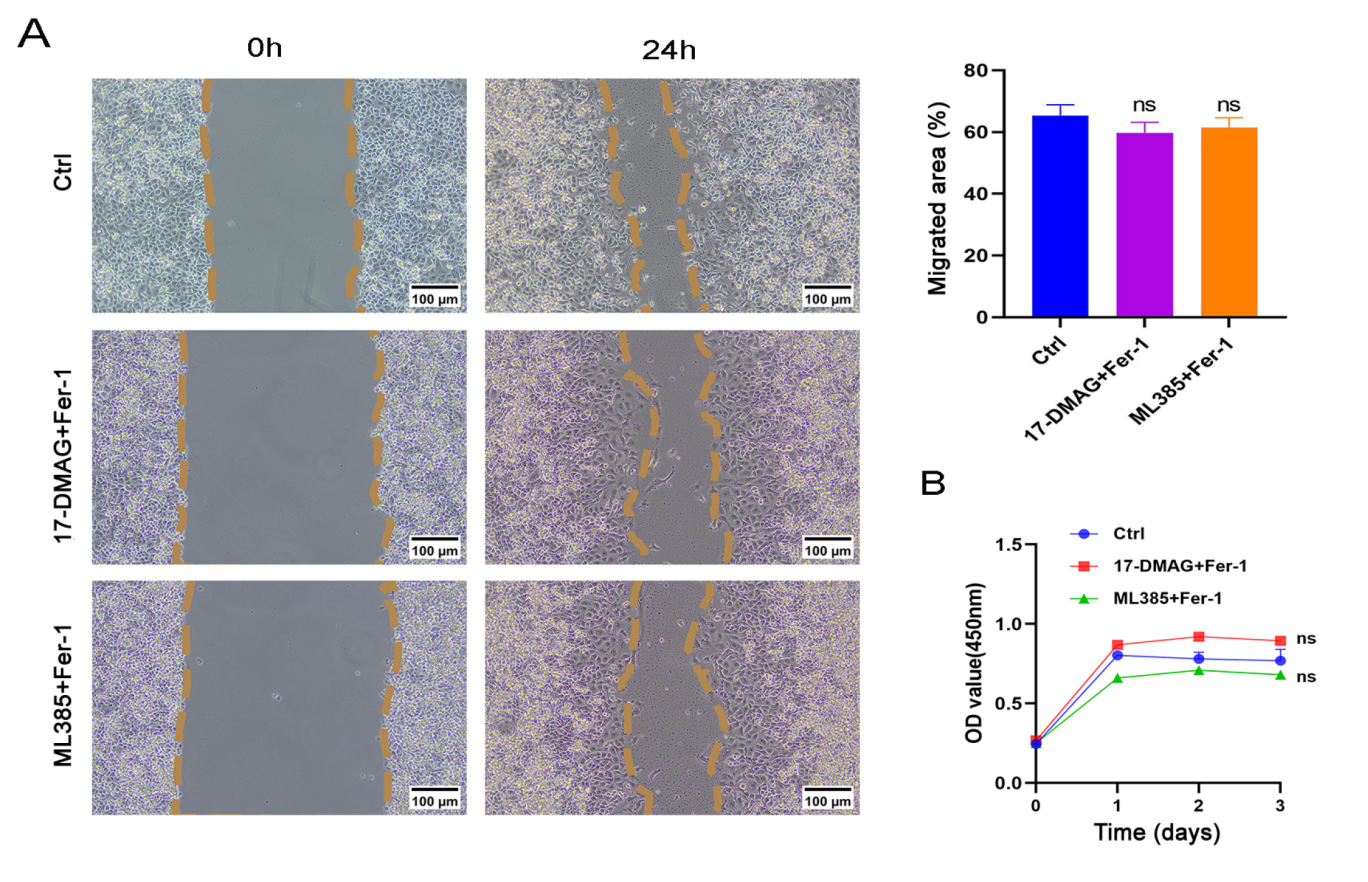
Figure 7 Synergistic effects of 17-DMAG, ML385, and Fer-1 in rescuing cell viability and proliferation (A) Wound healing assay of Ctrl, 17-DMAG + Fer-1 and ML385 + Fer-1 groups. Scale bar: 100 μm. (B) CCK8 assay of Ctrl, 17-DMAG + Fer-1 and ML385 + Fer-1 groups. Data are presented as the mean ± SD.

We performed cell rescue experiments in the present study. First, 50 nM 17-DMAG and 0.25 μM of Fer-1 ( Supplementary Figure S3D) were added to Panc-1-gem cells. The groups included the 17-DMAG + Fer-1 group, the ML385 + Fer-1 group, and the Ctrl group. Wound healing assays revealed that cell viability was improved in the 17-DMAG + Fer-1 and ML385 + Fer-1 groups, but the decrease in migration ability was not obvious ( Figure 7A). The results of the CCK-8 assay revealed that the 17-DMAG + Fer-1 group and the ML385 + Fer-1 group exhibited a rebound in cell proliferation ( Figure 7B).

## Discussion

Chemotherapy prolongs survival time, improves quality of life, and enhances the prognosis of patients with PC [13]. The most commonly used first-line treatment options for PC include gemcitabine-based monotherapy or combination chemotherapy. However, many patients with PC eventually develop chemoresistance, resulting in less favorable clinical outcomes. Therefore, a deeper understanding of the mechanisms underlying chemoresistance in PC is important.

Hsp90α is a highly conserved protein and a vital chaperone for cell survival and growth [14]. Recent studies have reported that Hsp90α can promote the migration of PC cells and enhance PC metastasis [15]. However, the effect of Hsp90α on the chemoresistance of PC remains poorly understood. We investigated patients with PC who received chemotherapy and revealed that the level of Hsp90α was significantly greater in the peripheral blood of patients with chemoresistance ( *n*  = 53) than in those of chemosensitive patients ( *n *= 65). Consistently, higher level of Hsp90α was detected in the peripheral blood of the same patients in the chemoresistance phase than in the chemosensitive phase. Additionally, we performed immunohistochemical analysis on tissue samples from chemosensitive PCs, adjacent noncancerous tissues, chemoresistant PCs with liver metastasis, and adjacent liver tissues. Our analysis revealed no significant differences in Hsp90α expression between chemosensitive cancer tissues and adjacent tissues, whereas significantly higher expression level of Hsp90α was detected in chemoresistant metastatic PC tissues than in adjacent tissues. On the basis of these findings, we hypothesized that Hsp90α plays a key role in inducing chemoresistance in PC. To validate this hypothesis, we utilized a Panc-1-gem cell line. Both western blot analysis and qRT-PCR confirmed that Hsp90α was upregulated in this model. Furthermore, we quantitatively assessed Hsp90α expression in the culture supernatant of Panc-1-gem cells and 4 other PC cell lines (Panc-1, Aspc-1, BxPc-3, and SW1990) using ELISA and observed significantly greater expression in the Panc-1-gem cell supernatant than in the other cell lines. To further investigate the role of Hsp90α, we introduced the Hsp90α inhibitor 17-DMAG into the culture medium of Panc-1-gem cells. The results demonstrated that compared with the control group, the 17-DMAG group exhibited reduced migration, invasion, and proliferation capabilities and increased apoptosis. Additionally, we performed siRNA interference with Hsp90α in Panc-1-gem cells and established control (Ctrl), siCtrl, and siHsp90α groups. We observed reduced cell proliferation, increased apoptotic cell death, and decreased invasion ability in the siHsp90α group compared with those in the control and siCtrl groups. Furthermore, we conducted *in vivo* experiments, including the Ctrl + gem, shCtrl + gem, and shHsp90α + gem groups. Tumor cells were subcutaneously inoculated into nude mice, and gemcitabine was intraperitoneally administered after tumor formation. Tumor volume and weight were periodically measured, and western blot analysis was performed. Compared with the Ctrl group, the shCtrl group presented no significant changes in tumor volume, whereas the shHsp90α group presented reduced tumor volume and weight. These results strongly indicated that Hsp90α plays a role in the development and progression of chemoresistance in PC.

To investigate the role of Hsp90α in promoting chemoresistance in PC cells, we performed a comparative RNA sequencing analysis between Panc-1 and Panc-1-gem cells. Our results revealed a significant increase in the expressions of ferroptosis-related proteins, including GPX4, SLC7A11, and SLC3A2, in the Panc-1-gem group, indicating a significant difference in the ferroptosis pathway between the two groups. Ferroptosis is a novel mode of cell death characterized by intracellular accumulation of ROS, iron overload, decreased glutathione (GSH) levels, and inactivation of GPX4 [ 16, 17]. Previous studies have demonstrated that inhibition of ARF6 enhances RSL3-induced ferroptosis, thereby ameliorating the chemoresistance of PC cells [18]. We subsequently added 5 μM erastin to the Panc-1-gem cell culture medium to induce ferroptosis and used the untreated group as the control group. The results revealed that adding erastin to Panc-1-gem cells increased intracellular iron level but weakened the migration and invasion abilities of cells and increased apoptosis. Moreover, transmission electron microscopy revealed that the morphology of the intracellular mitochondria changed, the mitochondria were wrinkled in Panc-1-gem cells, and the membrane became dense after the addition of erastin. These findings support our hypothesis that ferroptosis is suppressed in Panc-1-gem cells. We conducted further experiments to investigate the relationship between Hsp90α and ferroptosis inhibition in Panc-1-gem cells. First, we employed lentiviral infection to knock down *Hsp90α* in Panc-1-gem cells, establishing shHsp90α and shCtrl groups. ELISA results revealed significantly increased intracellular levels of iron and lipid ROS in the shHsp90α group compared with those in the shCtrl group. Additionally, western blot analysis revealed decreased expression of GPX4 and increased expression of ACSL4 in the shHsp90α group. Transmission electron microscopy also revealed notable alterations in the morphology of mitochondria in the shHsp90α group, suggesting the occurrence of ferroptosis. We also introduced 17-DMAG, an Hsp90α inhibitor, into the culture medium of Panc-1-gem cells to establish the 17-DMAG and Ctrl groups. Western blot analysis revealed a marked decrease in GPX4 expression in the 17-DMAG group. Furthermore, ELISA demonstrated increased intracellular concentrations of iron and lipid ROS in the 17-DMAG group. Collectively, these findings strongly suggested that Hsp90α facilitates chemoresistance in PC by impeding the occurrence of ferroptosis in drug-resistant strains. To further investigate the relationship between ferroptosis inhibition and Hsp90α, we used lentiviral infection to knock down *Hsp90α* in Panc-1-gem cells. This yielded two groups: the shHsp90α group and the shCtrl group. ELISA results revealed a significant increase in the intracellular levels of iron and lipid ROS in the shHsp90α group compared with those in the shCtrl group. Western blot analysis revealed decreased GPX4 expression and increased ACSL4 expression in the shHsp90α group. Moreover, transmission electron microscopy revealed alterations in mitochondrial morphology, suggesting the occurrence of ferroptosis in cells lacking Hsp90α. Additionally, we treated Panc-1-gem cells with 17-DMAG, an Hsp90α inhibitor, and established 17-DMAG and Ctrl groups. Western blot analysis revealed significantly decreased intracellular expression of GPX4 in the 17-DMAG group. Furthermore, ELISA revealed increased intracellular concentrations of iron and lipid ROS in the 17-DMAG group. Taken together, our results provided compelling evidence that Hsp90α can inhibit ferroptosis in Panc-1-gem cells.

Furthermore, we explored the inhibitory effect of ferroptosis on drug-resistant strains by targeting Hsp90α. According to previous reports, GPX4 plays a crucial role in ferroptosis, and Nrf2 regulates GPX4 expression [ 19, 20]. *GPX4* is a target gene of *Nrf2*, which is a major transcription factor for endogenous antioxidants, promotes cell survival and maintains cellular redox homeostasis [21]. Activation of the Nrf2 pathway has been shown to hinder ferroptosis [ 22, 23]. In our study, we used 17-DMAG to inhibit the expression of Hsp90α in Panc-1-gem cells. Western blot analysis revealed that 17-DMAG suppressed Nrf2 expression. To further validate our findings, we utilized the Nrf2 inhibitor ML385 and siRNA interference. The Panc-1-gem cells treated with the Nrf2 inhibitor were divided into two groups: the Ctrl group and the ML385 group. siRNA interference experiments were used to establish the siCtrl and siNrf2 groups. Our findings demonstrated that the ML385 group presented increased intracellular iron concentrations and decreased migration and invasion. Compared with the siCtrl group, the siNrf2 group had decreased cell proliferation, migration, and invasion but increased apoptosis. These findings suggest that Nrf2 inhibition in Panc-1-gem cells promotes ferroptosis. Thus, Hsp90α likely inhibits ferroptosis in drug-resistant strains by upregulating Nrf2, thereby promoting chemoresistance in PC. After the Panc-1 and Panc-1-gem groups were established for immunofluorescence co-localization, the results demonstrated the cytoplasmic localization of Hsp90α, while Nrf2 was mainly localized in the nucleus. Interestingly, Panc-1-gem cells presented the highest fluorescence intensities for Hsp90α in the cytoplasm and for Nrf2 in the nucleus, followed by Panc-1 cells. The 17-DMAG group presented the weakest fluorescence intensity. In addition, Nrf2 expression was most prominent in the nucleus of Panc-1-gem cells, whereas it was predominant in the cytoplasm of Panc-1 and 17-DMAG cells. Similarly, immunohistochemical staining of tumor tissues from nude mice revealed the cytoplasmic distribution of Nrf2 in the shHsp90α group and the nuclear localization of Nrf2 in the shCtrl group. According to previous reports, Keap1, a negative regulator of Nrf2, can prevent the nuclear translocation of Nrf2 to maintain the cellular redox status [24]. Keap1 anchors Nrf2 in the cytoplasm under normal physiological conditions. Keap1 serves as a substrate for the Cul3-dependent E3 ubiquitin ligase complex, promoting the ubiquitination and subsequent degradation of Nrf2 by the proteasome. However, reactive oxygen species or electrophilic attacks lead to the release of Nrf2 from Keap1 sequestration. Nrf2 is subsequently translocated into the nucleus [ 25, 26]. Nrf2 in Panc-1-gem cells was found to fluoresce more intensely in the nucleus via immunofluorescence. These results suggest that Nrf2 translocates to the nucleus when pancreatic cancer cells become drug resistant. Furthermore, Western blot analysis revealed high expression levels of Hsp90α and Keap1 in the cytoplasm and high level of Nrf2 in the nucleus of Panc-1-gem cells. Co-IP revealed the protein expressions of Hsp90α and KEAP1 in both the Input and IP groups, but Nrf2 protein expression was not detected in the IP group. On the basis of these observations, we propose that cytoplasmic Hsp90α in Panc-1-gem cells prevents Nrf2 sequestration from Keap1. Nrf2 subsequently translocates into the nucleus and enhances downstream gene expression. These alterations suppress ferroptosis and promote or exacerbate drug resistance in cancer cells. We also conducted rescue experiments. Panc-1-gem cells were rescued by the addition of an Hsp90 inhibitor or Nrf2 inhibitor followed by a ferroptosis inhibitor. Wound healing assays revealed that the migration ability of the 17-DMAG + Fer-1 and ML385 + Fer-1 groups was restored. The results of the CCK-8 assay revealed that, compared with that in the Ctrl group, cell proliferation was restored in the 17-DMAG + Fer-1 and ML385 + Fer-1 groups. Rescue experiments revealed that after inhibiting the expressions of Hsp90α and Nrf2 in Panc-1-gem cells, Fer-1, a ferroptosis inhibitor, restored the cell state.

In conclusion, this study revealed that Hsp90α plays a crucial role in promoting chemoresistance in PC. Mechanistically, Hsp90α liberates Nrf2 from Keap1 sequestration and promotes the nuclear translocation of Nrf2, which enhances Nrf2-mediated GPX4 expression and suppresses ferroptosis in chemoresistant PC cells. In other words, Hsp90α inhibits ferroptosis in PC cells through the Nrf2/GPX4 axis, thereby leading to chemoresistance. This study provides a potential target for enhancing the efficacy of chemotherapy in patients with PC.

## Supporting information

2410Supplementary_Figures

## Supplementary Data

Supplementary data is available at *Acta Biochimica et Biophysica Sinica* online.
