# Supplementary material for: Hsp90α promotes chemoresistance in pancreatic cancer by regulating Keap1-Nrf2 axis and inhibiting ferroptosis: Hsp90α promotes chemoresistance in PC
Source: Acta Biochim Biophys Sin (Shanghai). 2024 Aug 22;57(2):295–309. doi: 10.3724/abbs.2024138 (PMC11868932; doi:10.3724/abbs.2024138)
Supplement: 2410Supplementary_Figures [file 2410Supplementary_Figures.pdf]

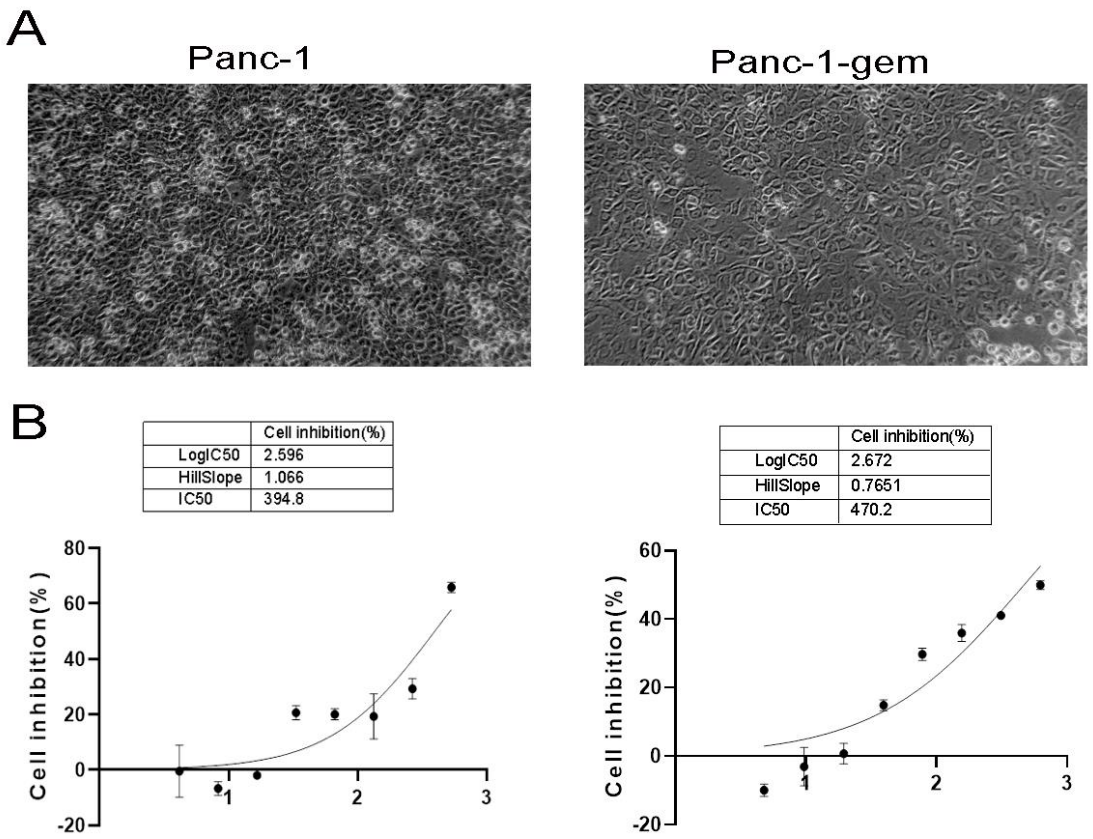

Supplementary Figure S1. Microscopic cellular morphology of Panc-1 and Panc-1-Gem groups and IC50 plot for gemcitabine resistance (A) The morphology of Panc-1 and Panc-1-gem cells observed by microscopy (×100). (B) The IC50 values of gemcitabine after 24 h in Panc-1 and Panc-1-gem cells.

Supplementary Figure S2. 17-DMAG concentration screening, validation of Hsp90a siRNA and shHsp90 efficacy (A) CCK8 was used to detect the effect of different concentrations of 17-DMAG on Panc-1-gem proliferation. (B) Western blot analysis was used to detect the expression of Hsp90α in 17-DMAG and Ctrl groups. (C) qRT-PCR was used to detect the levels of relative mRNA expression for *Hsp90* in Con, siCtrl, siHsp90α-1, siHsp90α-2, and siHsp90α-3 groups. (D) Western blot analysis of Hsp90 proteins in Con, siCtrl, siHsp90 α-1, siHsp90 α-2, and siHsp90 α-3 groups. (E) Fluorescence map of 0.1 μL Hsp90α-sh1 interference using lentiviral vector after 72 h of infection with 293T. (F) Flow cytometry used to compare apoptosis rates of 293T and shHsp90α-1 groups. Data are shown as the mean ± SD. **P<0.01, ***P<0.001.

Supplementary Figure S2. Concentration screening of Erastin, ML385 and Fer-1 (A)

CCK8 was used to detect the effect of different concentrations of Erastin on the proliferation of Panc-1-gem cells. (B) Western blot analysis of the effect of 5 μm and 10 μm of Erastin on GPX4 protein expression in Panc-1-gem cells. (C) CCK8 was used to detect the effect of different concentrations of ML385 on the proliferation of Panc-1-gem cells. (D) CCK8 was used to detect the effect of different concentrations of Fer-1 on the proliferation of Panc-1-gem cells. Data are displayed as the mean ± SD. Ns, *P*>0.05; **P*<0.05, ***P*<0.01, ****P*<0.001, *****P*<0.0001.

Supplementary Table S1 xxxx
